# Comprehensive evaluation of otorhinolaryngological symptoms in COVID-19 patients

**DOI:** 10.1186/s43163-022-00263-5

**Published:** 2022-06-08

**Authors:** Mohammad Salah Mahmoud, Mohamed Shehata Taha, Ossama Ibrahim Mansour, Michael Fadel, Ossama Mustafa Mady, Ahmed Abdelmoneim Teaima

**Affiliations:** Otorhinolaryngology Department, Faculty of Medicine, Ain Shams University, Cairo, Egypt

**Keywords:** COVID-19, SARS-CoV-2, Anosmia, Cough, Sore throat

## Abstract

**Introduction:**

The aim of this study is to comprehensively evaluate the incidence and natural course of otorhinolaryngological symptoms of COVID-19 infection and its relations to each other and patient’s demographics.

**Methods:**

This is a prospective study conducted on symptomatic adult patients proven to be infected with COVID-19. Detailed history was taken from each patient including onset of symptoms. Symptoms were followed up tightly. We focus on otorhinolaryngological (ORL) symptoms and their duration and onset in relation to other symptoms. Data were collected and analyzed in detail.

**Results:**

Six-hundred eighty-six patients were included in the study, their age ranged from 19-75 years old, and of them 55.1% were males. Cough was found in 53.1% of cases followed by sore throat in 45.8%, anosmia/ hyposmia in 42.3%, headache in 42%, rhinorrhea in 19.5%, dry mouth in 7.6%, globus in 6.1%, epistaxis in 4.4%, and hearing loss in 0.6%. In non-ORL symptoms, fever was found in 54.2%, malaise in 55.1%, dyspnea in 49.3%, and diarrhea in 27.2%. The first symptom was anosmia in 15.7% of cases, sore throat in 6.1 %, cough in 7.9%, and headache in 13.4% of cases. Fever was the first symptom in 22.7%, malaise in 25.1%, and diarrhea in 6.4%. Headache occurred for 5.5 ± 2 days, anosmia/hyposmia 3 to > 30 days, sore throat 4.1 ± 1.2 days, rhinorrhea 4.3 ± 1.1, cough 7.4 ± 2.5 days, fever 4.7 ± 2 days, and malaise 6.5 ± 2.4 days. The cluster of COVID-19-related symptoms showed nine principal components.

**Conclusion:**

Otorhinolaryngological symptoms are main symptoms in COVID-19 infection, and they should be frequently evaluated to detect suspected cases especially in pauci-symptomatic patients and to properly manage infected patients.

**Supplementary Information:**

The online version contains supplementary material available at 10.1186/s43163-022-00263-5.

## Background

The severe acute respiratory syndrome coronavirus 2 (SARS-CoV-2) began in Wuhan, China, at the end of 2019 [1]. In February 2020, it was officially called “COVID-19” by the WHO [2]. It became pandemic and has disastrous impact on health care and economic systems around the world. As of 20th April 2021, the WHO reports that 140 million people have been diagnosed with COVID-19 worldwide, with 3 million deaths, including 220 countries and territories. And mostly, the disease will continue to spread due to the reduction in strict measures [2, 3].

Seven coronaviruses are known to transmit to humans including SARS-CoV-2, SARS-CoV, and MERS-CoV (Middle East respiratory syndrome coronavirus) [4]. Growing evidence confirmed the nasal cavity is a vital area susceptible to SARS-CoV-2 infection. Researchers compared the pathology and virology of SARS-CoV-2, SARS-CoV, and MERS-CoV. They confirmed that these pathogenic coronaviruses have different mainly pathogenic sites: SARS-CoV-2 (nose and throat), SARS-CoV (lung), and MERS-CoV (type-2 pneumocytes). Viral loads in the patient’s nasal cavity were higher than the viral loads in the pharynx, both symptomatic individuals and asymptomatic ones, hinting the nasal cavity as the first gateway for the initial infection [5, 6].

COVID-19 symptoms are very similar to seasonal flu with the most common symptoms of fever, cough, shortness of breath, malaise, muscle aches, sore throat, headache, and tiredness, loss of taste, and or smell [7]. COVID-19 could be manifested by several symptoms, ranging from asymptomatic/mild symptoms to severe illness and death [8]. ENT manifestations are not uncommon symptoms of COVID-19, especially in mild or moderate form of the disease [9].

Mapping the symptoms and percentages of the disease will assist to discover COVID-19 disease more and create more effective treatment protocols. The general symptoms were discussed in detail in the literature, but to the best of our knowledge, there is a gap in detailed research on ENT manifestations in COVID-19 in the literature. So, here we collect the data from 686 patients confirmed to be COVID-19-positive by PCR, and this is the largest and most detailed case series study in the literature about this topic.

## Methods

This prospective study was conducted at the Ain Shams University Specialized Hospital at El-Obour City (assigned as a quarantine hospital during the pandemic for COVID-19 patients) from 1st May 2020 to 15th July 2020. This study included symptomatic adult patients proven to be infected with COVID-19 by PCR in nasopharyngeal swab. Exclusion criteria were asymptomatic patients, patients with mental or physical defects hindering communications, and patients with previous significant upper airway surgeries. Severely infected patients, who were admitted to intensive care unit, were excluded from the study. Lost patients in follow-up were excluded from the study.

Detailed history was taken from each patient personally by physicians or nurses completing sheets including demographic data, risk factors for COVID-19, comorbidities, and onset of symptoms. Symptoms were followed up tightly until complete recovery, or either patient was hospitalized or discharged for home isolation (followed by phone call). We focus on otorhinolaryngological (ORL) symptoms and their duration and onset in relation to other symptoms. ORL symptoms included in our study were cough, sore throat, anosmia, headache, nasal discharge, nasal obstruction, dysphagia, postnasal discharge, expectoration, dry mouth, earache, globus, vertigo, epistaxis, sneezing, tinnitus, facial pressure, otorrhea, stridor, deafness, neck swelling, and facial weakness. Non-ORL symptoms were fever, malaise, myalgia, diarrhea, and dyspnea.

### Statistical methods

Data were analyzed using IBM© SPSS© Statistics version 26 (IBM© Corp., Armonk, NY). Continuous numerical variables are presented as mean and standard deviation and categorical variables as counts and percentages.

Associations are examined using the Phi (ϕ) coefficient (for nominal-nominal association), rank biserial (rrb) correlation coefficient (for ordinal-nominal association), or point biserial (rpb) correlation coefficient (for continuous-nominal association).

We conducted maximum likelihood principal component analysis (PCA) on the 27 symptoms that we screened for. The Kaiser-Meyer-Olkin (KMO) measure of sampling adequacy and Bartlett’s test of sphericity were used to examine suitability of data for component analysis. A KMO value of ≥ 0.6 and a *P*-value < 0.05 for the Bartlett’s test were identified as criteria for sampling adequacy and feasibility of PCA.

Initial component extraction was based on a cutoff criterion of eigenvalue greater than 1. Besides, a scree plot was examined, and all components with eigenvalues situated on the sharp descent of the plot before it levelled out were retained. Based on the results of initial factor extraction, 9 principal components were rotated using an orthogonal (Varimax) rotation solution.

Two-sided *P*-values < 0.05 are considered statistically significant.

## Results

**Fig. 1:**
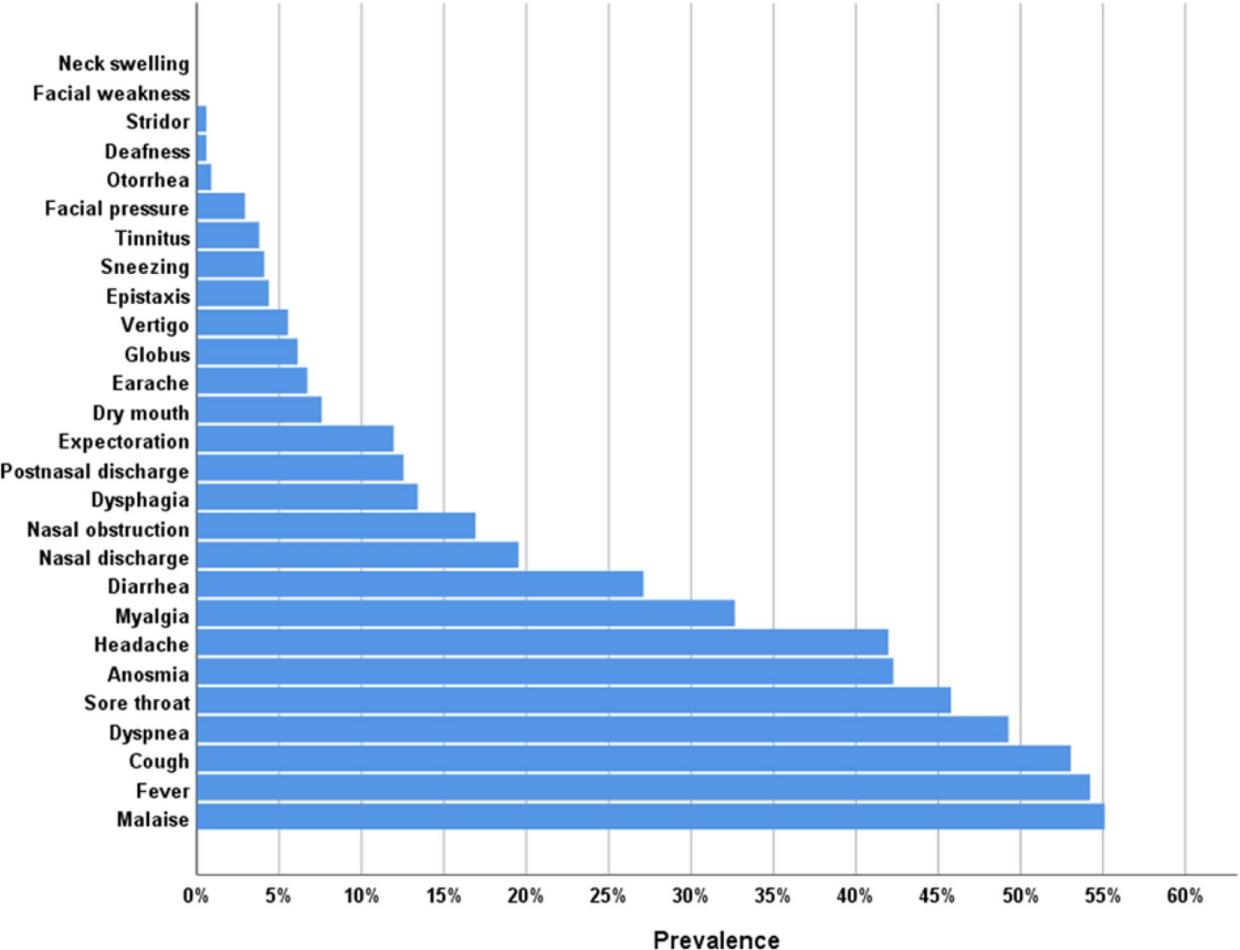
Prevalence of symptoms (both ORL and non-ORL) in COVID-19 patients

**Fig. 2:**
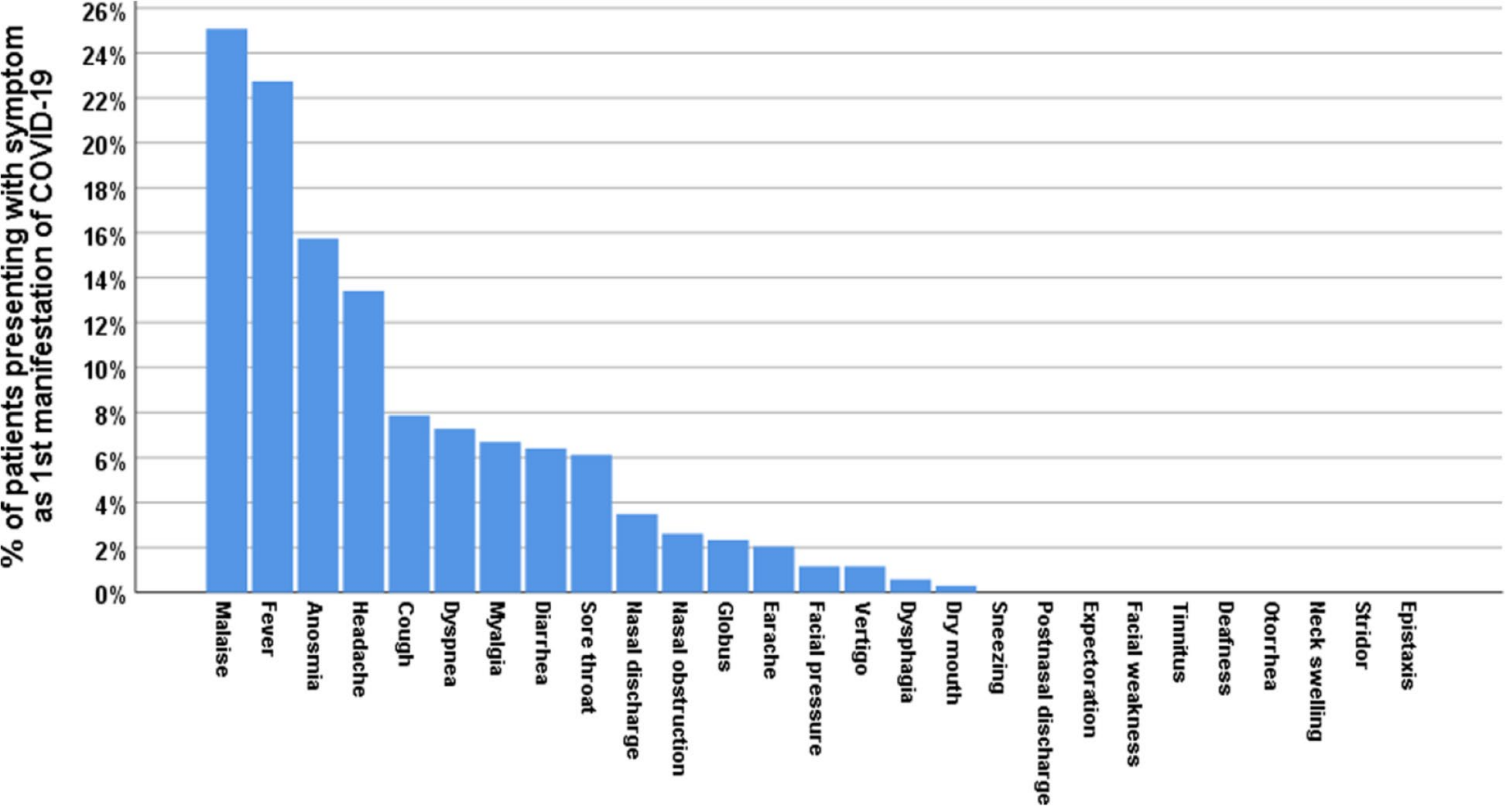
Percentage of patients presenting with various symptoms (ORL, non-ORL) as first manifestation of COVID-19

There were 686 patients included in our study aged 19 to 75 years old with 72% of them at or above 50 years old. A total of 65% were symptomatic before confirmation of diagnosis by PCR. A total of 82% were infected mildly (no need for hospitalization) (Table 1). Cough, sore throat, anosmia, and headache were the most prevalent ORL symptoms (Table 2) (Fig. 2), while non-ORL symptoms were presented as follows: malaise 55.1%, fever 54.2%, dyspnea 49.3%, myalgia 32.7%, and diarrhea 27.2% (Fig. 1).

**Table 1:** Characteristics of the study population

Variable		Count	%
**Age category**^**a**^	≤ 50 years	494	72.0%
	> 50 years	192	28.0%
**Sex**	F	308	44.9%
	M	378	55.1%
**Smoking**	Nonsmoker	472	68.8%
	Ex-smoker	68	9.9%
	Current smoker	146	21.3%
**Risk factors for COVID-19**	HCW	228	33.2%
	First responder	136	19.8%
	Close contact	246	35.9%
	Travel	4	0.6%
	Other risk factors	12	1.7%
**Number of risk factors for COVID-19**	Nil	196	28.6%
	I	370	53.9%
	II	104	15.2%
	III	16	2.3%
**Comorbidities**	Hypertension	148	21.6%
	Asthma	64	9.3%
	DM	124	18.1%
	COPD	24	3.5%
	CVS	20	2.9%
	Sinonasal chronic disease	64	9.4%
	Other comorbidities	50	7.3%
**Number of comorbidities**	Nil	354	51.9%
	I	196	28.7%
	II	116	17.0%
	III	6	0.9%
	IV	4	0.6%
	V	6	0.9%
**Onset of symptoms**	Pre-diagnosis	448	65.3%
	Post-diagnosis	238	34.7%
**Source of infection**	Unidentified	334	48.7%
	Identified	352	51.3%
**COVID-19 severity**	Mild	563	82.1%
	Moderate	123	17.9%

^a^Mean ± SD (minimum to maximum) = 42.3 ± 13.1 (19 to 75) years

**Table 2:** Prevalence of otorhinolaryngological symptoms in COVID-19 patients in order of decreasing frequency

Symptom	Count	%
**Cough**	364	53.1%
**Sore throat**	314	45.8%
**Anosmia**	290	42.3%
**Headache**	288	42.0%
**Nasal discharge**	134	19.5%
**Nasal obstruction**	116	16.9%
**Dysphagia**	92	13.4%
**Postnasal discharge**	86	12.5%
**Expectoration**	82	12.0%
**Dry mouth**	52	7.6%
**Earache**	46	6.7%
**Globus**	42	6.1%
**Vertigo**	38	5.5%
**Epistaxis**	30	4.4%
**Sneezing**	28	4.1%
**Tinnitus**	26	3.8%
**Facial pressure**	20	2.9%
**Otorrhea**	6	0.9%
**Stridor**	4	0.6%
**Deafness**	4	0.6%
**Neck swelling**	0	0.0%
**Facial weakness**	0	0.0%

**Fig. 3:**
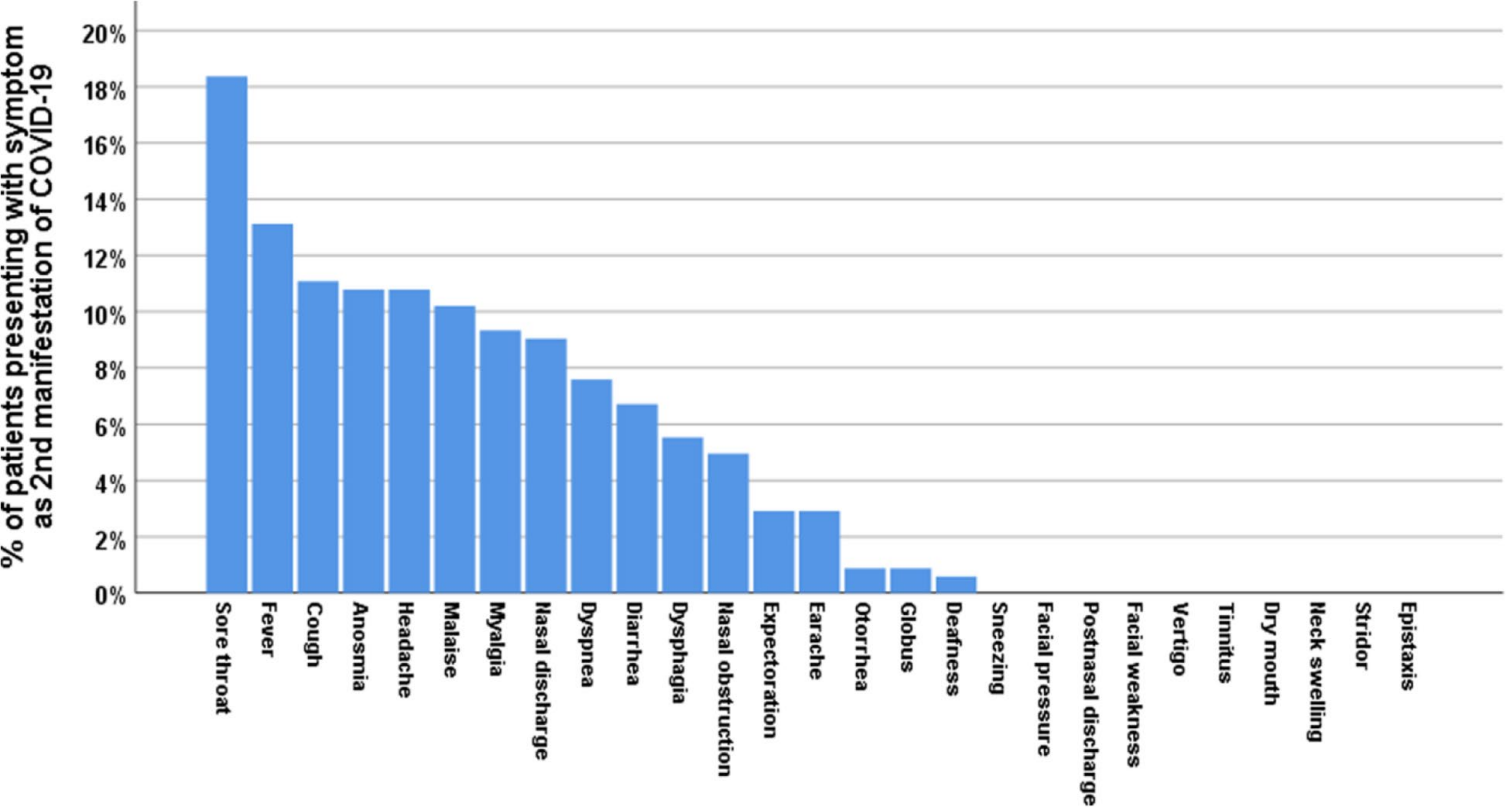
Percentage of patients presenting with various symptoms (ORL, non-ORL) as second manifestation of COVID-19

**Fig. 4:**
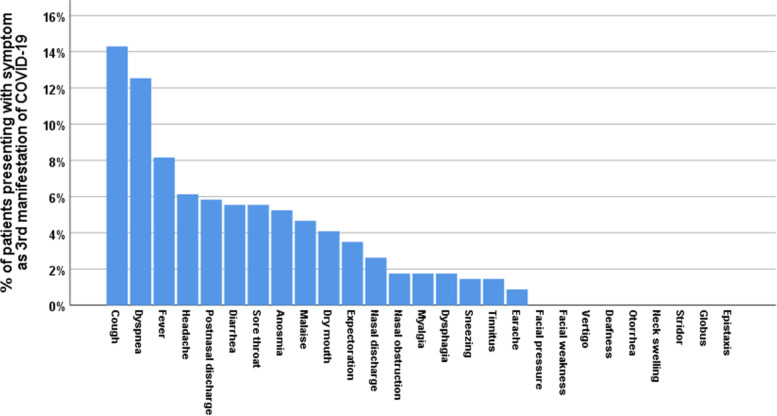
Percentage of patients presenting with various symptoms (ORL, non-ORL) as third manifestation of COVID-19

**Fig. 5:**
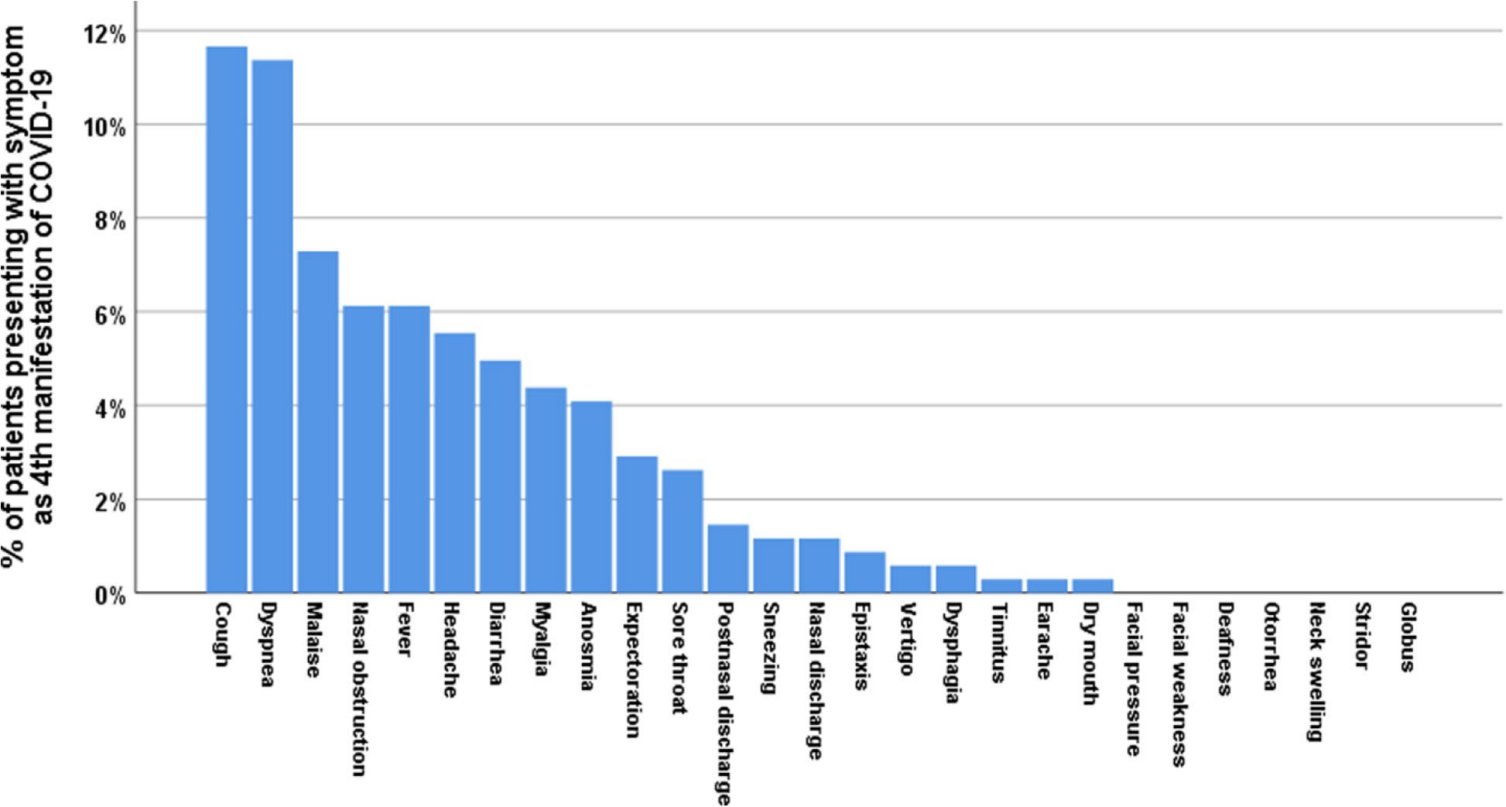
Percentage of patients presenting with various symptoms (ORL, non-ORL) as fourth manifestation of COVID-19

Headache presented as first symptom in 13.4% and anosmia in 15.7%. Sore throat presented as second symptom in 18.4%, cough in 11.1%, anosmia in 10.8%, and nasal discharge in 9% (Table 3) (Figs. 2, 3, 4, 5)

**Table 3:** Chronological order of ORL symptoms in COVID-19 patients

Symptom	Order of symptom in disease manifestation	Count	%
**Headache**	I	92	13.4%
	II	74	10.8%
	III	42	6.1%
	IV	38	5.5%
**Nasal obstruction**	I	18	2.6%
	II	34	5.0%
	III	12	1.7%
	IV	42	6.1%
**Nasal discharge**	I	24	3.5%
	II	62	9.0%
	III	18	2.6%
**Postnasal discharge**	III	40	5.8%
	IV	10	1.5%
	V	16	2.3%
**Sneezing**	III	10	1.5%
	IV	8	1.2%
**Anosmia**	I	108	15.7%
	II	74	10.8%
	III	36	5.2%
	IV	28	4.1%
	V	38	5.5%
**Sore throat**	I	42	6.1%
	II	126	18.4%
	III	38	5.5%
	V	26	3.8%
	VII	56	8.2%
**Dysphagia**	I	4	0.6%
	II	38	5.5%
	III	12	1.7%
	V	16	2.3%
**Cough**	I	54	7.9%
	II	76	11.1%
	III	98	14.3%
	IV	80	11.7%
	V	28	4.1%
**Dry mouth**	I	2	0.3%
	III	28	4.1%
	V	8	1.2%
**Earache**	I	14	2.0%
	II	20	2.9%
**Tinnitus**	III	10	1.5%
	IV	2	0.3%
**Vertigo**	I	8	1.2%
	V	14	2.0%
**Expectoration**	II	20	2.9%
	III	24	3.5%
	IV	20	2.9%

In non-ORL symptoms, fever presented as first symptom in 22.7% and second in 13.1%. Malaise presented as first symptom in 25.1% and second in 10.2%. Dyspnea presented as third symptom in 12.5% and fourth in 11.4%. Diarrhea presented as first symptom in 6.4% (Figs. 2, 3, 4, 5).

In relation to the duration of the symptom, anosmia took 13.4 ± 7.6 days, cough 7.4 ± 2.5 days, fever 4.7 ± 2 days, malaise 6.5 ± 2.4 days, and sore throat 4.1 ± 1.2 days (Supplemental Table 1).

By applying the principal component analysis for the cluster of COVID-19-related symptoms, it showed that groups of symptoms tend to be together. Group 1 symptoms are fever, headache, malaise, sore throat, cough, and dyspnea. Group 2 symptoms are nasal obstruction, nasal discharge, anosmia, etc. (Supplemental Table 2).

In terms of the association between the clinical symptoms and patient factors, as regards age, there was a statistically significant association between younger age and fever and cough. As regards gender, there was a statistically significant association between male gender and epistaxis. As regards smoking, there was a statistically significant association between smoking and headache, malaise, and sore throat. As regards asthma, there was a statistically significant association between asthma and malaise and post-nasal discharge. As regards diabetes mellitus, there was a statistically significant association between DM and fever, malaise, sneezing, and anosmia. As regards COPD, there was a statistically significant association between COPD and nasal obstruction, nasal discharge, epistaxis, dysphagia, and expectoration (Supplemental Table 3)

## Discussion

COVID-19 disease rapidly spreads across every corner of the world. Otorhinolaryngologists may be in the front line due to the close contact with the mucus membrane of the upper respiratory tract, so discussing ORL symptoms in COVID infection in details may help in knowing the pauci-symptomatic patients. Our study primarily is concerned with ORL symptoms of COVID-19 infection. It is done through our pandemic hospital on symptomatic patients confirmed to be infected by PCR. Six-hundred eighty-six patients were included in our study aged 42.3 ± 13.1 (19 to 75) years old, with 55.1% were male. In our study, close contact with an infected person formed the main risk factor in 35.9% of cases. About 28.6% of cases had no identified risk factor for infection. About 51.9% of cases included had no comorbidities. Most of the cases (65.3%) were symptomatic before diagnosis by PCR, and 51.3% of cases had identified source of infection. Most of the cases (82.1%) were mild COVID-19, and the rest were moderate to severe.

To date, our study may be the most detailed in the literature concerning ORL symptoms in COVID-19 patients, including duration of each symptom, its onset, relation to other symptoms, and patients’ demographics. Out of 686 patients, cough was the most prevalent ORL symptom found in 364 patients (53.1%) followed by sore throat in 314 (45.8%), anosmia and hyposmia in 290 (42.3%), headache in 288 (42%), rhinorrhea in 134 (19.5%), nasal obstruction in 116 (16.9%), and dry mouth in 52 (7.6%). Earache was found in 46 (6.7%), globus in 42 (6.1%), epistaxis in 30 (4.4%), tinnitus in 26 (3.8%), and hearing loss in 4 (0.6%). In non-ORL symptoms, fever was found in 372 (54.2%), malaise in 378 (55.1%), dyspnea in 388 (49.3%), myalgia in 224 (32.7%), and diarrhea in 186 (27.2%).

In their study on 1099 COVID-19 patients, Guan et al. found cough in 67.8%, dyspnea in 18.7%, sore throat in 13.9, and nasal obstruction in 4.8% [10]. Lechien et al. (in their study on 417 patients) found fever in 48%, cough in 78%, anosmia/hyposmia in 85.6%, and headache in 46% [11]. Speth et al. found fever in 88.5%, anosmia/hyposmia 61.2%, cough in 61.1%, and headache in 12.1% [12]. Bhatta et al. (in their study on 600 patients) documented sore throat in 88%, fever in 78.8%, anosmia/hyposmia in 63.6%, rhinorrhea in 51.3%, nasal obstruction in 33.5%, sneezing in 30.3%, dyspnea in 18.6%, cough in 87%, headache in 82%, myalgia in 67%, and diarrhea in 14.3% [13].

In 138 patients, Wang et al. found that common symptoms were fever (99%), fatigue (70%), dry cough (59%), anorexia (40%), myalgia (35%), dyspnea (3%), and expectoration (27%) [14]. In 155 patients, Elibol found that cough is (43.8%), anosmia (35.4%), sore throat (27%), nasal obstruction (12.9%), postnasal discharge (6%), otalgia (2%), rhinorrhea (9%), tinnitus (1.2%), hearing loss (0.6%). A total of 58.7% of the 155 patients were female, and 42.2% were male. ORL symptoms were seen more in females compared to males [15]. Lee et al. found anosmia in 15.3% of their patient group [16].

Borah et al. (in their study on 2000 patients) stated percentage of symptoms as follows: sore throat 80%, headache 76%, hyposmia/anosmia 44%, nasal obstruction 28%, fever 93%, cough 85%, dyspnea 33%, malaise 14%, and abdominal symptoms 4% [17]. Ozcelik et al. (in their study on 116 patients) found that dry cough is (53.4%), dyspnea (38.8%), headache (37%) and nausea/ vomiting (31%), hyposmia/anosmia (37.9%), sore throat (32.7%), dysphagia (20.6%), globus sensation (13.7%), nasal obstruction (27.5%), rhinorrhea (13.7%), sneezing (12.9%), tinnitus (11.2%), hearing impairment (5.2%), vertigo (6.1%) [18].

Based on our study, the most common ORL symptoms are cough, sore throat, and anosmia/hyposmia, while the least common was hearing loss in four patients, and there were no cases of neck swelling or facial paralysis. It is not clear whether these four patients were symptomatic of the disease or were seen as an incidental finding to the disease.

In terms of the chronological order of ORL symptoms, anosmia/ hyposmia was the first symptom in 15.7% of cases, second in 10.8%, and third in 5.2%. Sore throat was the first symptom in 6.1% of cases, second in 18.4%, and third in 5.5%. Cough was the first symptom in 7.9% of cases, second in 11.1%, third in 14.3%, and fourth in 11.7%. Headache was the first symptom in 13.4% of cases, second in 10.8%, and third in 6.1%. Rhinorrhea was the second symptom in 9% of cases. Postnasal discharge was the third symptom in 5.8% of cases. In non-ORL symptoms, fever presented as first symptom in 22.7% and second in 13.1%. Malaise presented as first symptom in 25.1% and second in 10.2%. Dyspnea presented as third symptom in 12.5% and fourth in 11.4%. Diarrhea presented as first symptom in 6.4%.

In terms of duration of each ORL symptoms, headache occurred for 5.5 ± 2 (2–14) days, anosmia/hyposmia 13.4 ± 7.6 (3 to > 30) days, sore throat 4.1 ± 1.2 (2–10) days, rhinorrhea 4.3 ± 1.1 (2–7) days, cough 7.4 ± 2.5 days, and dry mouth 4.7 ± 1.6. In non-ORL symptoms, fever occurred for 4.7 ± 2 (1–14) days, malaise 6.5 ± 2.4 (3–17) days, diarrhea 5.1 ± 2.6 (2–14) days, and dyspnea 8.6 ± 5.5 (4 to > 30) days.

According to Borah et al., ORL symptoms of 78% of cases resolved in 10–20 days, but anosmia/hyposmia might need some more time [17], while Bhatta et al. found the time required for symptom resolution was for dyspnea (1.3 ± 0.2 days), fever (2.4 ± 0.9 days), anosmia/hyposmia (14.3 ± 2.8 days), nasal obstruction (10.1 ± 3.1 days), sneezing (8.3 ± 1.3 days), sore throat (7.6 ± 2.3 days), and rhinorrhea (6.3 ± 1.2 days) (13). Teaima et al. found smell and taste dysfunctions happened before other COVID-19 symptoms in 19.4% of cases, with other COVID-19 symptoms in 37.1% and after in 43.5%. These dysfunctions occurred suddenly in 80.4% and gradually in 19.6%. In 6-month follow-up, 66% of smell/taste symptoms recovered completely, 22.1% partially, while 11.9% did not recover [19]. Mahmoud et al. found that 50.6% of hospitalized COVID-19-infected patients complained of olfactory/gustatory dysfunctions, and 76.5% of them complained upper respiratory tract symptoms before olfactory/gustatory symptoms [20].

Principal component analysis for the cluster of COVID-19-related symptoms shows 9 principal components. Fever, headache, malaise, sore throat, cough, and dyspnea formed one component. Nasal obstruction, rhinorrhea, postnasal discharge, and anosmia/hyposmia formed one component. Dry mouth and myalgia formed one component. Dysphagia, diarrhea, and globus formed one component. Vertigo, tinnitus, and hearing loss formed one component.

Ozcelik et al. found that cough and dyspnea were more common in elder patients (over 60 years), and that anosmia/hyposmia, headache, and sore throat were common in younger patients and in female. Anosmia/hyposmia and sneezing were more common in patients with allergic rhinitis, while headache was more common in hypertensive and diabetic patients. Dyspnea was more common in patient with chronic chest diseases [18].

In our study, we found a statistically significant association between younger age and fever cough and association between male gender and epistaxis. As regards smoking, there is a statistically significant association between smoking and headache, malaise, and sore throat and a statistically significant association between asthma and malaise and post-nasal discharge. As regards diabetes mellitus, there is a statistically significant association between DM and fever, malaise, sneezing, and anosmia. As regards COPD, there was a statistically significant association between COPD and nasal obstruction, nasal discharge, epistaxis, dysphagia, and expectoration.

Limitations of this study are that 72% of included patients were at or above 50 years old, and that we could not follow-up symptoms in severely infected patients in intensive care unit.

## Conclusion

Otorhinolaryngological symptoms are main symptoms in COVID-19 infection, and they should be frequently evaluated to detect suspected cases especially in pauci-symptomatic patients and to properly manage infected patients.

## Supplementary Information

**Additional file 1: Supplemental Table 1.** Chronological pattern (duration) of symptoms (ORL, non-ORL) in COVID-19 patients.

**Additional file 2: Supplemental Table 2.** Results of principal component analysis for the cluster of COVID-19-related symptoms showing symptom loadings on the 9 principal components extracted and rotated using an orthogonal (Varimax) rotation solution.

**Additional file 3: Supplemental Table 3.** Association between symptoms and demographic and epidemiological variables.

## Data Availability

All data generated or analyzed during this study are included in this article. Further inquiries can be directed to the corresponding author.
