# Supplementary material for: Comprehensive evaluation of otorhinolaryngological symptoms in COVID-19 patients
Source: Egypt J Otolaryngol. 2022 Jun 8;38(1):73. doi: 10.1186/s43163-022-00263-5 (PMC9175524; doi:10.1186/s43163-022-00263-5)
Supplement: Supplementary file 1 — Additional file 1: Supplemental Table 1. Chronological pattern (duration) of symptoms (ORL, non-ORL) in COVID-19 patients. [file 43163_2022_263_MOESM1_ESM.docx]

**Supplemental Table 1. Chronological pattern (duration) of symptoms (ORL, non-ORL) in COVID-19 patients**

|  | Duration (days) | | | | | | |
| --- | --- | --- | --- | --- | --- | --- | --- |
| Symptom | Mean | SD | Minimum | Maximum | 25^th^ Percentile | Median | 75^th^ Percentile |
| Fever | 4.7 | 2.0 | 1.0 | 14.0 | 4.0 | 5.0 | 5.0 |
| Headache | 5.5 | 2.0 | 2.0 | 14.0 | 4.0 | 5.0 | 7.0 |
| Malaise | 6.5 | 2.4 | 3.0 | 17.0 | 5.0 | 6.0 | 7.0 |
| Nasal obstruction | 4.0 | 1.4 | 2.0 | 7.0 | 3.0 | 3.0 | 5.0 |
| Nasal discharge | 4.3 | 1.1 | 2.0 | 7.0 | 4.0 | 4.0 | 5.0 |
| Postnasal discharge | 5.1 | 1.6 | 3.0 | 10.0 | 4.0 | 5.0 | 6.0 |
| Facial pressure | 5.2 | 1.3 | 4.0 | 7.0 | 4.0 | 5.0 | 7.0 |
| Sneezing | 3.9 | 1.1 | 2.0 | 5.0 | 3.0 | 4.0 | 5.0 |
| Anosmia | 13.4 | 7.6 | 3.0 | 30.0 | 8.0 | 12.0 | 15.0 |
| Epistaxis | 1.7 | 0.9 | 1.0 | 3.0 | 1.0 | 1.0 | 3.0 |
| Sore throat | 4.1 | 1.2 | 2.0 | 10.0 | 3.0 | 4.0 | 5.0 |
| Dysphagia | 3.8 | 1.2 | 2.0 | 6.0 | 3.0 | 4.0 | 5.0 |
| Globus | 4.5 | 1.3 | 2.0 | 7.0 | 3.0 | 5.0 | 5.0 |
| Cough | 7.4 | 2.5 | 3.0 | 14.0 | 5.0 | 7.0 | 10.0 |
| Stridor | 1.0 | 0.0 | 1.0 | 1.0 | 1.0 | 1.0 | 1.0 |
| Dry mouth | 4.7 | 1.6 | 3.0 | 7.0 | 3.0 | 5.0 | 7.0 |
| Earache | 4.3 | 1.1 | 2.0 | 6.0 | 3.0 | 5.0 | 5.0 |
| Otorrhea | 3.0 | 0.0 | 3.0 | 3.0 | 3.0 | 3.0 | 3.0 |
| Deafness | 5.0 | 0.0 | 5.0 | 5.0 | 5.0 | 5.0 | 5.0 |
| Tinnitus | 3.9 | 1.2 | 2.0 | 5.0 | 3.0 | 5.0 | 5.0 |
| Vertigo | 4.1 | 1.4 | 2.0 | 6.0 | 2.0 | 5.0 | 5.0 |
| Dyspnea | 8.6 | 5.5 | 4.0 | 30.0 | 5.0 | 7.0 | 10.0 |
| Diarrhea | 5.1 | 2.6 | 2.0 | 14.0 | 3.0 | 5.0 | 5.0 |
| Expectoration | 5.9 | 1.9 | 3.0 | 10.0 | 4.0 | 5.0 | 7.0 |
| myalgia | 6.4 | 3.0 | 3.0 | 20.0 | 4.0 | 5.0 | 8.0 |

SD = standard deviation.
