# Supplementary material for: Comprehensive evaluation of otorhinolaryngological symptoms in COVID-19 patients
Source: Egypt J Otolaryngol. 2022 Jun 8;38(1):73. doi: 10.1186/s43163-022-00263-5 (PMC9175524; doi:10.1186/s43163-022-00263-5)
Supplement: Supplementary file 2 — Additional file 2: Supplemental Table 2. Results of principal component analysis for the cluster of COVID-19-related symptoms showing symptom loadings on the 9 principal components extracted and rotated using an orthogonal (Varimax) rotation solution. [file 43163_2022_263_MOESM2_ESM.docx]

**Supplemental Table 2. Results of principal component analysis for the cluster of COVID-19-related symptoms showing symptom loadings on the 9 principal components extracted and rotated using an orthogonal (Varimax) rotation solution**

|  | Component | | | | | | | | |
| --- | --- | --- | --- | --- | --- | --- | --- | --- | --- |
| Symptom | 1 | 2 | 3 | 4 | 5 | 6 | 7 | 8 | 9 |
| Fever | **0.578** | -0.150 | 0.065 | 0.416 | 0.119 | 0.197 | 0.076 | 0.116 | 0.192 |
| Headache | **0.798** | 0.046 | -0.056 | -0.208 | -0.035 | 0.080 | -0.103 | 0.033 | 0.129 |
| Malaise | **0.448** | 0.273 | 0.094 | 0.349 | -0.443 | 0.078 | -0.096 | 0.065 | -0.180 |
| Nasal obstruction | -0.126 | **0.850** | 0.024 | -0.059 | 0.023 | 0.090 | 0.003 | -0.016 | -0.038 |
| Nasal discharge | 0.044 | **0.805** | 0.085 | -0.046 | 0.076 | -0.008 | 0.197 | -0.083 | -0.069 |
| Postnasal discharge | -0.182 | **0.670** | -0.089 | 0.291 | 0.086 | -0.083 | -0.008 | -0.116 | 0.179 |
| Facial pressure | 0.132 | 0.077 | -0.024 | -0.115 | -0.042 | -0.013 | **0.680** | 0.142 | 0.517 |
| Sneezing | 0.075 | 0.090 | -0.065 | 0.170 | -0.106 | 0.032 | 0.046 | 0.076 | **0.761** |
| Anosmia | 0.083 | **0.569** | -0.074 | -0.031 | -0.270 | -0.111 | -0.229 | 0.043 | 0.187 |
| Epistaxis | 0.095 | 0.140 | -0.019 | -0.122 | **0.661** | 0.061 | -0.155 | 0.240 | -0.045 |
| Sore throat | **0.478** | 0.425 | 0.286 | 0.119 | 0.038 | -0.308 | -0.080 | 0.108 | 0.173 |
| Dysphagia | 0.489 | 0.106 | 0.035 | -0.031 | 0.133 | 0.081 | 0.022 | **0.633** | 0.068 |
| Globus | -0.059 | -0.094 | -0.017 | -0.055 | 0.153 | -0.052 | 0.003 | **0.741** | 0.199 |
| Cough | **0.734** | -0.155 | 0.226 | 0.165 | 0.187 | -0.148 | 0.108 | -0.026 | 0.023 |
| Stridor | -0.005 | 0.007 | -0.007 | 0.034 | 0.049 | -0.016 | **0.881** | -0.048 | -0.053 |
| Dry mouth | -0.055 | -0.148 | 0.093 | **0.652** | 0.131 | -0.067 | -0.163 | 0.000 | 0.258 |
| Earache | 0.085 | 0.099 | **0.775** | -0.110 | -0.051 | 0.127 | -0.024 | -0.137 | -0.010 |
| Otorrhea | -0.057 | -0.079 | **0.787** | 0.033 | -0.044 | -0.071 | 0.023 | 0.033 | -0.032 |
| Deafness | -0.111 | -0.043 | 0.181 | -0.006 | -0.125 | **0.779** | 0.002 | -0.005 | 0.087 |
| Tinnitus | 0.049 | 0.025 | **0.659** | 0.081 | 0.234 | **0.529** | -0.037 | 0.068 | -0.049 |
| Vertigo | 0.194 | -0.019 | -0.082 | -0.081 | 0.397 | **0.704** | -0.025 | -0.062 | -0.061 |
| Dyspnea | **0.767** | -0.088 | -0.139 | 0.024 | 0.053 | -0.005 | 0.087 | 0.022 | -0.105 |
| Diarrhea | 0.030 | -0.194 | -0.139 | 0.304 | -0.222 | -0.062 | -0.002 | **0.611** | -0.345 |
| Expectoration | 0.204 | -0.074 | 0.042 | 0.295 | **0.699** | 0.097 | 0.263 | -0.049 | -0.120 |
| Myalgia | 0.126 | 0.283 | -0.152 | **0.697** | -0.131 | -0.029 | 0.135 | 0.022 | -0.064 |

Two symptoms (Neck swelling & Facial weakness) were not included as neither is reported by any of the patients.

Two symptoms (Facial pressure & Sore throat) have rather equal loading on 2 components.
