# Supplementary material for: Comprehensive evaluation of otorhinolaryngological symptoms in COVID-19 patients
Source: Egypt J Otolaryngol. 2022 Jun 8;38(1):73. doi: 10.1186/s43163-022-00263-5 (PMC9175524; doi:10.1186/s43163-022-00263-5)
Supplement: Supplementary file 3 — Additional file 3: Supplemental Table 3. Association between symptoms and demographic and epidemiological variables. [file 43163_2022_263_MOESM3_ESM.docx]

**Supplemental Table 3. Association between symptoms and demographic and epidemiological variables**

|  |  | Symptom | | | | | | | | | | | | | | | | | | | | | | | | |
| --- | --- | --- | --- | --- | --- | --- | --- | --- | --- | --- | --- | --- | --- | --- | --- | --- | --- | --- | --- | --- | --- | --- | --- | --- | --- | --- |
| Variable | **Association measure** | **Fever** | **Headache** | **Malaise** | **Nasal obstruction** | **Nasal discharge** | **Postnasal discharge** | **Facial pressure** | **Sneezing** | **Anosmia** | **Epistaxis** | **Sore throat** | **Dysphagia** | **Globus** | **Cough** | **Stridor** | **Dry mouth** | **Earache** | **Otorrhea** | **Deafness** | **Tinnitus** | **Vertigo** | **Dyspnea** | **Diarrhea** | **Expectoration** | **Myalgia** |
| Age | ***r_pb_*** | .141** | 0.051 | 0.046 | 0.007 | 0.029 | -.095* | -0.023 | 0.025 | -.173** | 0.036 | -0.046 | -0.025 | -0.015 | .147** | .112** | -0.030 | .105** | -0.045 | 0.062 | 0.016 | 0.031 | .165** | -0.075 | .123** | 0.002 |
|  | ***P*** | <0.001 | 0.186 | 0.232 | 0.853 | 0.453 | 0.013 | 0.554 | 0.506 | <0.001 | 0.343 | 0.224 | 0.513 | 0.688 | <0.001 | 0.003 | 0.427 | 0.006 | 0.236 | 0.102 | 0.671 | 0.414 | <0.001 | 0.051 | 0.001 | 0.960 |
| Male sex | ***φ*** | 0.018 | -0.064 | -0.049 | 0.063 | 0.060 | 0.041 | -.087* | .097* | -0.034 | .136** | -.100** | -0.006 | -0.038 | -0.003 | -.085* | .081* | 0.031 | .085* | -.085* | .087* | -0.012 | -.142** | 0.023 | .116** | -0.046 |
|  | ***P*** | 0.642 | 0.097 | 0.202 | 0.098 | 0.114 | 0.286 | 0.022 | 0.011 | 0.369 | <0.**001** | 0.009 | 0.876 | 0.315 | 0.930 | 0.026 | 0.033 | 0.416 | 0.026 | 0.026 | 0.023 | 0.753 | <0.001 | 0.545 | 0.002 | 0.225 |
| Smoking status | ***r_rb_*** | -.076* | -.148** | -.187** | 0.053 | .079* | 0.048 | -.090* | -.132** | -0.073 | .124** | -.167** | -0.033 | -0.015 | -0.068 | -0.002 | -0.022 | -0.029 | .169** | -0.049 | .133** | 0.063 | -.126** | -0.030 | 0.049 | -.142** |
|  | ***P*** | 0.048 | <0.**001** | <0.**001** | 0.168 | 0.038 | 0.214 | 0.019 | 0.001 | 0.058 | 0.001 | <0.**001** | 0.393 | 0.693 | 0.077 | 0.952 | 0.564 | 0.443 | <0.**001** | 0.201 | <0.**001** | 0.102 | 0.001 | 0.427 | 0.199 | <0.**001** |
| Ex-/Current Smoking | ***φ*** | -.089* | -.139** | -.227** | 0.049 | .129** | 0.030 | -.079* | -.139** | -.092* | .102** | -.139** | -0.043 | -0.041 | -0.060 | 0.031 | -0.050 | -0.004 | .140** | -0.052 | .130** | .085* | -.135** | -0.057 | .101** | -.133** |
|  | ***P*** | 0.020 | <0.**001** | <0.**001** | 0.202 | 0.001 | 0.431 | 0.038 | <0.**001** | 0.016 | 0.007 | <0.**001** | 0.256 | 0.287 | 0.115 | 0.416 | 0.189 | 0.908 | <0.**001** | 0.177 | 0.001 | 0.027 | <0.**001** | 0.137 | 0.008 | <0.**001** |
| Presence of risk factors for COVID-19 | ***φ*** | 0.015 | .080* | 0.065 | -0.042 | 0.067 | .200** | .110** | -.130** | 0.045 | .135** | .167** | 0.022 | 0.054 | 0.000 | 0.048 | .132** | 0.015 | 0.059 | 0.048 | .126** | .153** | 0.055 | 0.008 | .094* | 0.041 |
|  | ***P*** | 0.699 | 0.035 | 0.090 | 0.274 | 0.078 | <0.**001** | 0.004 | 0.001 | 0.241 | **<0.001** | **<0.001** | 0.571 | 0.159 | 1.000 | 0.205 | 0.001 | 0.700 | 0.120 | 0.205 | 0.001 | <0.**001** | 0.148 | 0.828 | 0.014 | 0.280 |
| Hypertension | ***φ*** | 0.055 | -0.001 | -0.025 | .085* | -0.062 | .080* | .246** | .143** | -0.004 | -.112** | -.098* | -0.040 | -0.045 | -0.075 | .146** | 0.010 | -.112** | -0.049 | -0.040 | -0.067 | -0.065 | 0.036 | -.097* | -0.040 | -.108** |
|  | ***P*** | 0.150 | 0.980 | 0.508 | 0.026 | 0.106 | 0.037 | **<0.001** | **<0.001** | 0.916 | 0.003 | 0.010 | 0.295 | 0.237 | 0.050 | <0.**001** | 0.784 | 0.003 | 0.197 | 0.293 | 0.080 | 0.089 | 0.346 | 0.011 | 0.292 | 0.005 |
| Asthma | ***φ*** | -.128** | -0.070 | .209** | -0.064 | -.082* | .302** | -0.056 | -0.066 | -.092* | -0.069 | -0.073 | -.126** | -.082* | 0.000 | -0.025 | 0.060 | -.086* | -0.030 | -0.025 | -0.064 | -.078* | 0.005 | 0.007 | .129** | .097* |
|  | ***P*** | 0.001 | 0.068 | <0.**001** | 0.092 | 0.031 | <0.**001** | 0.146 | 0.083 | 0.016 | 0.073 | 0.055 | 0.001 | 0.032 | 0.991 | 0.521 | 0.119 | 0.024 | 0.431 | 0.521 | 0.096 | 0.042 | 0.903 | 0.849 | 0.001 | 0.011 |
| DM | ***φ*** | .143** | -.108** | .211** | 0.031 | -0.021 | -0.041 | -.081* | .133** | -.157** | .159** | -.112** | 0.060 | 0.006 | .184** | -0.036 | -0.049 | -0.035 | .200** | -0.036 | .224** | .085* | .219** | .123** | .131** | .158** |
|  | ***P*** | <0.**001** | 0.005 | <0.**001** | 0.423 | 0.579 | 0.289 | 0.033 | **<0.001** | **<0.001** | **<0.001** | 0.003 | 0.118 | 0.866 | <0.001 | 0.347 | 0.203 | 0.359 | <0.001 | 0.347 | <0.001 | 0.026 | <0.001 | 0.001 | 0.001 | <0.001 |
| COPD | ***φ*** | .111** | .095* | 0.012 | .210** | .186** | -0.072 | -0.033 | -0.039 | -0.067 | .270** | -.079* | .251** | -0.049 | -0.043 | -0.015 | -0.055 | .076* | -0.018 | .402** | .461** | .578** | .130** | -0.009 | .174** | -0.065 |
|  | ***P*** | 0.004 | 0.013 | 0.746 | **<0.001** | **<0.001** | 0.059 | 0.388 | 0.304 | 0.081 | **<0.001** | 0.038 | **<0.001** | 0.203 | 0.256 | 0.703 | 0.154 | 0.047 | 0.640 | **<0.001** | **<0.001** | **<0.001** | 0.001 | 0.813 | <0.**001** | 0.089 |
| CVS | ***φ*** | -.084* | .133** | .156** | .245** | .221** | 0.039 | -0.030 | -0.036 | .097* | -0.037 | .189** | -0.017 | -0.044 | .163** | -0.013 | -0.050 | .439** | -0.016 | -0.013 | -0.034 | -0.042 | .176** | -.106** | .096* | -.121** |
|  | ***P*** | 0.027 | <0.001 | <0.001 | <0.001 | <0.001 | 0.307 | 0.432 | 0.350 | 0.011 | 0.332 | <0.001 | 0.650 | 0.247 | <0.001 | 0.729 | 0.194 | <0.001 | 0.670 | 0.729 | 0.368 | 0.272 | <0.001 | 0.006 | 0.012 | 0.002 |
| Sinonasal chronic disease | ***φ*** | .277** | .195** | .086* | .098* | 0.071 | 0.063 | -0.053 | 0.035 | .234** | -0.069 | .189** | .142** | -.082* | .121** | -0.025 | 0.059 | -.087* | -0.030 | -0.025 | -0.064 | -.078* | .246** | .096* | 0.067 | .096* |
|  | ***P*** | <0.001 | <0.001 | 0.024 | 0.010 | 0.062 | 0.100 | 0.167 | 0.364 | <0.001 | 0.072 | <0.001 | <0.001 | 0.031 | 0.002 | 0.519 | 0.123 | 0.024 | 0.429 | 0.519 | 0.095 | 0.041 | <0.001 | 0.012 | 0.082 | 0.012 |
| Number of comorbidities | ***r_rb_*** | .117** | 0.064 | .236** | .205** | .100** | .094* | 0.050 | .079* | 0.027 | 0.003 | -0.036 | 0.052 | -.108** | .086* | .107** | -0.021 | 0.043 | 0.029 | 0.023 | .094* | 0.046 | .274** | 0.007 | .162** | 0.015 |
|  | ***P*** | 0.002 | 0.096 | <0.001 | <0.001 | 0.009 | 0.014 | 0.192 | 0.039 | 0.487 | 0.942 | 0.354 | 0.172 | 0.005 | 0.025 | 0.005 | 0.581 | 0.257 | 0.455 | 0.543 | 0.014 | 0.231 | <0.001 | 0.865 | <0.001 | 0.696 |
| Presence of Comorbidities | ***φ*** | .136** | 0.074 | .202** | .119** | -0.041 | 0.014 | .098* | .097* | 0.033 | -0.006 | -.083* | -0.011 | -.100** | .164** | .080* | -0.033 | 0.045 | .098* | .080* | .146** | 0.048 | .214** | -0.036 | .131** | 0.027 |
|  | ***P*** | <0.001 | 0.053 | <0.001 | 0.002 | 0.288 | 0.709 | 0.011 | 0.012 | 0.395 | 0.873 | 0.031 | 0.772 | 0.009 | <0.001 | 0.037 | 0.386 | 0.237 | 0.011 | 0.037 | <0.001 | 0.214 | <0.001 | 0.349 | 0.001 | 0.487 |
| Post-diagnosis onset of symptoms | ***φ*** | 0.055 | -.124** | .116** | -.100** | -.096* | -.183** | -.126** | .128** | 0.046 | -.156** | -.129** | -.089* | 0.044 | -0.026 | -0.056 | 0.046 | .123** | -0.068 | -0.056 | -.113** | -0.069 | -.081* | .175** | -.174** | .250** |
|  | ***P*** | 0.151 | 0.001 | 0.002 | 0.009 | 0.011 | <0.001 | 0.001 | 0.001 | 0.231 | <0.001 | 0.001 | 0.020 | 0.252 | 0.492 | 0.144 | 0.231 | 0.001 | 0.073 | 0.144 | 0.003 | 0.069 | 0.033 | <0.001 | <0.001 | <0.001 |
| Identified source of infection | ***φ*** | -.134** | 0.001 | .153** | 0.019 | .112** | .157** | -.109** | -.094* | .161** | -.134** | 0.029 | -.130** | -0.068 | -.168** | -.079* | -0.030 | -.089* | -.096* | -.079* | -.143** | -.198** | -.078* | .135** | -.198** | .181** |
|  | ***P*** | <0.001 | 0.973 | <0.001 | 0.614 | 0.003 | <0.001 | 0.004 | 0.014 | <0.001 | <0.001 | 0.455 | 0.001 | 0.077 | <0.001 | 0.040 | 0.440 | 0.020 | 0.012 | 0.040 | <0.001 | <0.001 | 0.040 | <0.001 | <0.001 | <0.001 |
| Moderate severity of COVID-19 | ***φ*** | 0.056 | 0.018 | 0.025 | -0.018 | -0.029 | 0.007 | 0.032 | 0.038 | -0.008 | -0.026 | 0.013 | 0.061 | 0.023 | 0.036 | 0.014 | 0.010 | 0.042 | 0.038 | 0.064 | 0.047 | 0.020 | -0.005 | -0.037 | 0.039 | -0.009 |
|  | ***P*** | 0.145 | 0.634 | 0.519 | 0.633 | 0.448 | 0.862 | 0.404 | 0.320 | 0.841 | 0.503 | 0.735 | 0.108 | 0.543 | 0.346 | 0.712 | 0.800 | 0.274 | 0.324 | 0.094 | 0.224 | 0.606 | 0.905 | 0.331 | 0.312 | 0.805 |

*r_pb_* = point-biserial, *r_rb_* = rank-biserial, *φ* = phi coefficient of association, *P* = P-value.

* = significant at the *P*<0.05 level, ** = significant at the *P*<0.01 level.
